# Papillary Thyroid Carcinoma Concealed Within a Branchial Cyst Without Primary Thyroid Involvement: Unveiling the Enigma

**DOI:** 10.7759/cureus.61268

**Published:** 2024-05-28

**Authors:** Sahrish Khawaja, Maryam Arshad, Waqas Shafiq, Ahmed Imran Siddiqi

**Affiliations:** 1 Endocrinology, Shaukat Khanum Memorial Cancer Hospital and Research Centre, Lahore, PAK; 2 Endocrinology and Diabetes, Shaukat Khanum Memorial Cancer Hospital and Research Centre, Lahore, PAK

**Keywords:** thyroid cancer, lateral neck mass, fine-needle aspiration, thyroidectomy, branchial cleft cyst, papillary carcinoma of thyroid

## Abstract

Branchial cleft cysts are congenital anomalies that form during fetal development and originate from the second branchial cleft. They typically manifest as painless masses on the side of the neck and can become symptomatic when infected. These cysts can create a cavity that may foster infection and, in rare instances, facilitate the spread of primary tumors. It is unusual to find ectopic thyroid tissue within a brachial cyst and it is even rarer to see papillary thyroid carcinoma developing from this tissue. Whenever physicians find a case of lateral neck cyst containing thyroid neoplasm without a known primary in the thyroid, there is always a confusion about whether it is a case of metastatic disease with an undetected primary tumor, or is a carcinoma originating from ectopic thyroid tissue.

This is a case report of a papillary thyroid cancer that was unintentionally discovered inside a branchial cyst. So far, only five cases akin to this have been documented. There was no sign of an underlying primary thyroid tumor after the patient had a complete thyroidectomy and selected neck dissection, according to a comprehensive evaluation. This article touches on the development of thyroid tissue within branchial cysts and discusses the etiology of lateral neck tumors. The outcome for such patients appears to be favorable after cyst excision and total thyroidectomy. This article also emphasizes the importance of doing routine histopathological examinations on surgically removed samples that look benign.

## Introduction

Branchial cysts are the most prevalent lateral congenital neck masses that originate from the second branchial cleft [1-3]. It's important to note that most branchial cleft cysts are benign [1,4]. Most of them are discovered incidentally without any symptoms and are removed primarily for cosmetic reasons. Branchial cleft cysts can occasionally harbor ectopic thyroid tissues, and although quite rare, the development of papillary thyroid carcinoma (PTC) within this thyroid tissue is possible. In such instances, tumors are not typically found in the thyroid gland but are instead localized within the branchial cyst itself.

This case report highlights an incident where PTC was unexpectedly discovered within a branchial cleft cyst after excision. A comprehensive treatment approach was taken, involving a total thyroidectomy and selective neck dissection. Post-surgical histopathological examination revealed no signs of the hidden primary disease. This report briefly delves into the possible causes of these lateral neck masses and explores the potential presence of ectopic thyroid tissue in them. Additionally, it outlines management recommendations for lateral neck malignancies without a discernible primary source.

## Case presentation

A 20-year-old woman presented with a painless swelling in her neck for the last five months that was progressively increasing in size. There were no constitutional symptoms, dysphagia, dysphonia, or shortness of breath. She had no family history of thyroid cancer and was clinically euthyroid. Upon physical examination, a 2 cm mass was observed on the front of her neck inferior to the mandible, which did not move with swallowing and showed no cutaneous fistula. There was no stridor, no tracheal deviation, and no palpable lesions in the thyroid. Cervical lymph nodes were not palpable.

A neck ultrasound was conducted revealing a 2 cm complex cystic lesion in the upper anterior neck with a few internal septations. Additionally, there was a 1.1 cm heterogeneously echogenic mass in the lower part of the cyst, displaying tiny calcification foci. The differential diagnosis included branchial or dermoid cysts.

**Figure 1:**
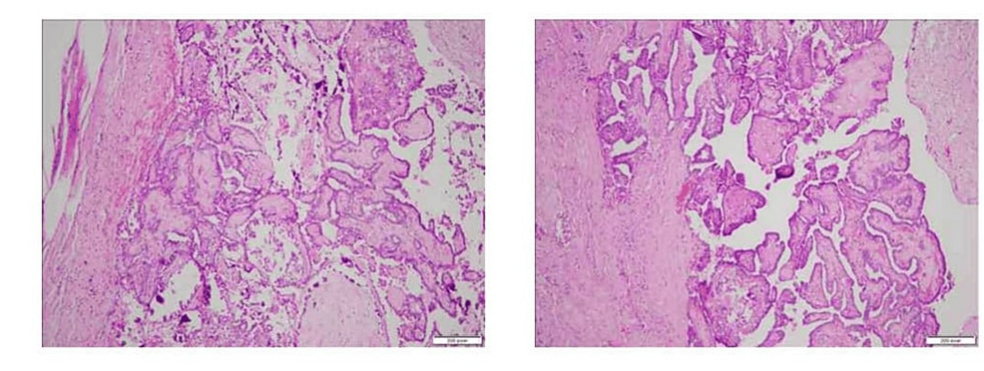
Histopathology images Histological examination of the section reveals fragments of tissue showing a malignant neoplasm arranged in papillary architecture. The individual neoplastic cells show nuclear elongation and enlargement, grooves, nuclear overcrowding, overlapping, and clearing. Psammoma bodies are also seen.

Subsequently, the patient underwent surgical excision of the cyst. Histopathological analysis reported a neoplasm arranged in a papillary architecture, characterized by cells with nuclear elongation, clearing, overlapping, and grooving of nuclei. Immunohistochemistry with thyroid transcription factor-1 (TTF-1) stain tested positive, consistent with classic papillary thyroid carcinoma (Figure 1). Background lymphoid tissue was absent, and the region around the PTC showed no pathological signs of normal thyroid tissue.

Magnetic resonance imaging of the neck was performed but the results were unremarkable, showing no discrete lesions or nodal disease. Considering the possibility of metastatic dissemination from a thyroid primary, a total thyroidectomy and selective neck dissection were carried out, with plans for radioactive iodine therapy afterward. Histopathology from this surgery revealed benign thyroid tissue with lymphocytic inflammation and no evidence of malignancy. Additionally, two reactive lymph nodes were identified. 

Following thyroidectomy, the patient was followed by an endocrinologist who started her on an appropriate dose of thyroxine to keep thyroid stimulating hormone suppressed. There were no postoperative complications. The patient was referred to the nuclear medicine department for radioactive iodine therapy.

## Discussion

To our current understanding, there have been only five documented instances of Papillary Thyroid Carcinoma (PTC) developing inside a branchial cyst without a thyroid primary [5-8]. There are some instances of PTC within lateral neck cysts, where either a primary thyroid tumor was later identified, or no thorough pathological examination of the thyroid was conducted. When a primary thyroid tumor is discovered in conjunction with PTC in a lateral neck cyst, the natural assumption is that it's due to metastatic spread. However, in cases where no primary is evident, we face a diagnostic conundrum: Does this represent a case of PTC arising from ectopic thyroid tissue within the lateral neck cyst, or is it a case of metastatic illness with an undiagnosed primary cancer? If it is indeed ectopic tissue within a lateral neck cyst, the question arises as to how it migrated there.

Branchial cleft cysts are the most prevalent form of cystic neck abnormalities found in the lateral region, typically manifesting in individuals during their fourth decade of life. Although these cysts often remain symptom-free, they can occasionally lead to intermittent swelling. In some cases, upper respiratory infections can prompt rapid growth, elevating the risk of secondary infection and airway blockage, which may drive patients to seek medical attention. The principal treatment for symptomatic branchial cysts involves surgical removal after a diagnostic assessment with computed tomography scans and ultrasound [9,10]. The examination of post-surgical specimens through histopathological and immuno-histochemical analyses is a common procedure. A tiny proportion of cases of branchial cleft cysts may have metastatic components despite the fact that the majority are benign.

A retrospective study revealed that out of 28 branchial cleft cysts observed at a single institution over eight years, three contained metastatic tissue among which 66% were papillary thyroid carcinoma [11]. PTCs are typical thyroid cancers that have the ability to spread. These tumors often present as asymptomatic congenital neck masses, with many being non-palpable "incidentalomas" that escape detection during routine thyroid examinations, only to be discovered inadvertently through imaging. Malignancy should be suspected in cases of rapid increase in the size of head and neck masses, masses with diameter greater than 1 cm, enlargement of cervical lymph nodes, vocal cord paralysis with hoarseness of voice, family history of cancer or a history of significant childhood exposure to radiation [12]. Nevertheless, overall survival can reach as high as 90% at the 10-year mark with early initiation of treatment [13]. 

The occurrence of papillary thyroid carcinoma (PTC) metastasis within a branchial cleft cyst has been documented in the literature, though it remains a rare phenomenon [14-17]. A comprehensive review of these cases unveiled a common pattern. In each instance, a patient underwent the removal of a branchial cleft cyst, only to have unexpected histological findings indicative of PTC. In each of the cases, the examination of the excised tissue ultimately led to the discovery of PTC. This case underscores the significance of routinely conducting histopathological examinations on seemingly benign surgically removed lesions. It's worth noting that the removal of a branchial cleft cyst is typically seen as an optional surgery when there are no symptoms and adverse consequences may arise from delayed evaluation.

Now the question arises: Do most centers perform fine needle aspiration cytology (FNAC) and ultrasound for cysts that appear benign? The answer is FNAC and imaging with ultrasound should be the first step in the work-up of a neck mass. In instances of pure cysts, FNAC may not be diagnostic since there are few and indefinable solid constituents. So, if a prior ultrasound shows a cyst to be a well-circumscribed round/ovoid anechoic mass with thin walls and posterior acoustic enhancement, then it is likely benign and needs to be simply observed. However, if the lesion has solid components, dystrophic calcifications, and appears ill-defined and thick-walled, with septae and heterogeneous internal echoes, then it should be differentiated from metastatic lymph nodes through FNAC.

Despite extensive research into branchial cysts, their origin remains elusive. Earlier theories suggested that branchial cleft cysts are congenital anomalies that occur due to the malfunction of the branchial pouch apparatus. However, more recent hypotheses propose that cystic degeneration brought on by the migration of epithelial inclusions into lymph nodes is the cause of the development of some lateral neck masses.

According to these latest theories, once epithelial tissue from the upper aerodigestive tract or glandular tissue enters a cervical lymph node through lymphatic channels, it triggers degeneration, resulting in the formation of a lateral cervical cyst [7]. Lymphatic tissue is frequently visible in the histopathology of these lateral cysts [8], this presents a difficulty in distinguishing between cystic lymph nodes and branchial cysts. This cystic transformation and the subsequent development of carcinoma might elucidate the pathophysiology in our particular case, and similar cases have been reported [5,7].

## Conclusions

Patients with carcinoma in lateral neck cysts, especially after total thyroidectomy without a primary site, show promising outcomes. Consensus recommends total thyroidectomy and selective neck dissection when encountering such cases, emphasizing the importance of comprehensive neck examination and imaging like ultrasound and CT scans to distinguish metastatic lymph nodes from branchial cysts because they might look alike. The presence of atypical features in cysts suggests complex pathology, warranting fine-needle aspiration cytology (FNAC). Given the potential presence of papillary microcarcinoma, it is advisable to conduct serial thin-section analyses of all embedded thyroid tissue blocks. This case report emphasizes the significance of performing regular histopathological examinations on surgical specimens, even when the lesions appear benign.

## References

[1] Goff CJ, Allred C, Glade RS (2012). Current management of congenital branchial cleft cysts, sinuses, and fistulae. Curr Opin Otolaryngol Head Neck Surg.

[2] Muller S, Aiken A, Magliocca K, Chen AY (2015). Second branchial cleft cyst. Head Neck Pathol.

[3] Acierno SP, Waldhausen JH (2007). Congenital cervical cysts, sinuses and fistulae. Otolaryngol Clin North Am.

[4] Thomaidis V, Seretis K, Tamiolakis D, Papadopoulos N, Tsamis Tsamis (2006). Branchial cysts. A report of 4 cases. Acta Dermatovenerol Alp Pannonica Adriat.

[5] Mremi A, Nkya G, Amsi P, Sadiq A, Lodhia J, Pallangyo A (2023). Papillary thyroid carcinoma arising from ectopic thyroid tissue in a neck branchial cyst. SAGE Open Med Case Rep.

[6] Mehmood RK, Basha SI, Ghareeb E (2006). A case of papillary carcinoma arising in ectopic thyroid tissue within a branchial cyst with neck node metastasis. Ear Nose Throat J.

[7] Sidhu S, Lioe TF, Clements B (2000). Thyroid papillary carcinoma in lateral neck cyst: missed primary tumour or ectopic thyroid carcinoma within a branchial cyst?. J Laryngol Otol.

[8] Regauer S, Gogg-Kamerer M, Braun H, Beham A (1997). Lateral neck cysts—the branchial theory revisited. A critical review and clinicopathological study of 97 cases with special emphasis on cytokeratin expression. APMIS J Pathol Microbiol Immunol.

[9] So YK, Kim MJ, Kim S, Son YI (2018). Lateral lymph node metastasis in papillary thyroid carcinoma: a systematic review and meta-analysis for prevalence, risk factors, and location. Int J Surg.

[10] Haugen BR, Alexander EK, Bible KC (2016). 2015 American Thyroid Association Management Guidelines for Adult Patients with Thyroid Nodules and Differentiated Thyroid Cancer: The American Thyroid Association Guidelines Task Force on Thyroid Nodules and Differentiated Thyroid Cancer. Thyroid.

[11] Yehuda M, Schechter ME, Abu-Ghanem N, Golan G, Horowitz G, Fliss DM, Abu-Ghanem S (2018). The incidence of malignancy in clinically benign cystic lesions of the lateral neck: our experience and proposed diagnostic algorithm. Eur Arch Otorhinolaryngol.

[12] Caron NR, Clark OH (2006). Papillary thyroid cancer. Curr Treat Options Oncol.

[13] Cooc A, Chong I, Wang KY, Jiang K, Lincolns CM (2020). Papillary thyroid carcinoma metastasis to a branchial cleft cyst: a case report and review of imaging. Clin Imaging.

[14] Gur H, Arpaci RB, Ismi O, Dag A, Vayisoglu Y, Gorur K (2019). Papillary thyroid carcinoma spreading into branchial cleft cyst. Turkish Arch Otorhinolaryngology.

[15] Tazegul G, Bozoğlan H, Doğan Ö, Sari R, Altunbaş HA, Balci MK (2018). Cystic lateral neck mass: thyroid carcinoma metastasis to branchial cleft cyst. J Cancer Res Ther.

[16] Papadakis C, Ladias A, Chimona T, Gavriilidis M, Zisoglou M, Proimos E (2017). Thyroid papillary carcinoma in a branchial cleft cyst—a case report. Journal of cancer research and therapeutics.

[17] A. K. Ohri, S. K. Ohri, and M. P. Singh (1994). Evidence for thyroid development from the fourth branchial pouch. J Laryngol Otol.

